# Vitamin D and Exercise Performance in Professional Soccer Players

**DOI:** 10.1371/journal.pone.0101659

**Published:** 2014-07-03

**Authors:** Nikolaos E. Koundourakis, Nikolaos E. Androulakis, Niki Malliaraki, Andrew N. Margioris

**Affiliations:** 1 Department of Clinical Chemistry, School of Medicine, University of Crete, Heraklion, Greece; 2 Department of Laboratory Haematology, University Hospital, Heraklion, Crete, Greece; 3 Departments of Clinical Chemistry, University of Crete, School of Medicine, and University Hospital, Heraklion, Greece; Charité, Campus Benjamin Franklin, Germany

## Abstract

**Aim:**

The current study had two aims. The primary purpose was to examine the association between serum vitamin D levels and the ergometric evaluation of muscle strength, aerobic capacity, and speed in professional soccer players. The secondary aim was to evaluate the effects of the soccer off-season period on serum vitamin D levels.

**Methods:**

Sixty-seven Caucasian male soccer players (age 25.6±6.2 and height 1.81±0.08 m), members of two Greek Superleague Soccer teams and one Football-league championship team participated in this study. Exercise performance testing for the determination of squat jump (SJ), countermovement jump (CMJ), 10 (10 m) and 20 meters (20 m) sprint performance, maximal oxygen consumption (VO_2_max), anthropometry, and blood sampling were performed before (pre) and after (post) the six-week off-season period.

**Results:**

Analysis of our results showed the following: (a) a significant correlations between serum vitamin D levels and performance parameters in both pre (SJ; P<0.001, CMJ; P<0.001, VO_2_max; P<0.001, 10 m; P<0.001, and 20 m; P<0.001) and post (SJ; P<0.001, CMJ; P<0.001, VO_2_max; P = 0.006, 10 m; P<0.001, and 20 m; P<0.001) experimental sessions. (b) Vitamin D concentration increased significantly (P<0.001) following the six-week off-season period compared to baseline, while at the same time all measured performance parameters decreased (SJ; P<0.001, CMJ; P<0.001, 10 m; P<0.001, 20 m; P<0.001, VO_2_max; P<0.001).

**Discussion:**

Our findings suggest that vitamin D levels are associated with the ergometric evaluation of muscle strength, as expressed by SJ and CMJ, sprinting capacity, and VO_2_max in professional soccer players, irrespective the levels of performance. Furthermore, our data reaffirm the importance of UVB on serum vitamin D levels. Moreover, reductions in exercise training stress may also have beneficial effects on vitamin D levels, suggesting a possible association of its levels and the training-induced stress. Our results indicate a possibly bidirectional interaction between soccer performance indices and vitamin D levels.

## Introduction

Vitamin D is primarily synthesized endogenously following cutaneous exposure to ultraviolet B radiation (UVB) [1], [2]. Apart from its effect on calcium homeostasis and bone metabolism vitamin D exerts a host of other physiological effects on neural and muscular tissues, the immune system, and energy homeostasis, thus affecting among other parameters physical performance [3], [4], [5]. More specifically, it has been shown that vitamin D levels correlate with grip and quadriceps strength, physical fitness, and a decline of falls and bone fractures [5], [6]. Vitamin D deficiency predominantly affects the weight-bearing antigravity muscles of the lower limbs which are necessary for walking and postural balance [5], [7]. Furthermore, vitamin D supplementation boosts muscular strength and restores balance [7].

It should be noted that the majority of the above mentioned studies have been performed in the elderly [5], [7]. Nevertheless, similar findings have been reported in younger individuals. A recent study on adolescent girls reported a positive association between serum vitamin D levels and jump height, jump velocity, and power [2]. Similarly, early studies on collegiate athletes and students have documented that cardiovascular fitness, muscle endurance, and speed are enhanced following exposure to ultraviolet radiation [8], although other authors failed to document such associations [9]. Furthermore, a consisted literature indicates that physical and athletic performance is seasonal, it peaks when vitamin D levels peaks and declines as its levels decline [10], [11].

Paradoxically, a growing number of studies report a high prevalence of vitamin D insufficiency or downright deficiency even in regions with extensive sunlight in both athletic and non-athletic populations [12], [13]. The reason for this phenomenon is not entirely clear. It is mainly attributed to the limited exposure to sun, the types of clothing, and the declining ability of the skin to produce vitamin D precursors with advancing age [4]. Interestingly, a recent study reported that vitamin D levels also decline during strenuous military training [14]. This decrease was evident although training was performed outdoors in the summer and early autumn months during daylight hours and thus with adequate exposure under UVB. The latter data may indicate a possible relationship between exercise training stress and vitamin D levels.

Soccer is a sport where aerobic capacity, muscular strength and speed are of vital importance for most of the actions during playing. Indeed, players must perform repeated sprints, stops, jumps, and changes of direction with maximal force development, and in the shortest possible response time. Furthermore, top level players run approximately 10–12 km during a soccer game, and the total distance covered is linearly related to VO_2_max [15]. Vitamin D seems to be involved on this type of activities. It is well documented that its levels are related with muscle strength [5], the proportion and the diameter of type II muscle fibers [16], and neuromuscular coordination [12], which are of paramount importance for explosive type human movements, such as sprints and jumps [17], [18], [19]. Moreover, the presence of vitamin D receptors (VDR) in the muscle cell appears to influence muscle strength. The discovery of VDR within the muscle indicates that vitamin D has the potential to impact upon muscle structure and function directly and has been identified as a regulator of skeletal muscle development and action [20]. In regard to aerobic capacity the findings from recent studies provide evidence that it could be affected by vitamin D levels through an effect of this vitamin on optimal lung function and/or erythropoietin resistance [21], [22].

Our study had two aims. The main objective was to examine the potential relationship between vitamin D levels and muscle strength, as expressed by squat jump (SJ) and countermovement jump (CMJ) [23], maximal oxygen consumption (VO_2_max), 10 and, 20 meters sprint performance in two different occasions, prior to the beginning and at the end of the off-season soccer period. Our second aim was to examine the vitamin D response to the reduced exercise training during the six-week off-season transition period. We hypothesized that in both experimental sessions vitamin D levels would correlate with soccer players’ jumping, sprinting, and aerobic capacity, and that the off-season transition period, of reduced training stress, would favorably affect vitamin D concentration. To the best of our knowledge, no published study has examined the relationship between vitamin D levels and muscle strength, VO_2_max and speed in professional soccer players, and/or the effects off-season detraining soccer period on its levels in any kind of athletic population.

## Materials and Methods

### Participants

Initially, seventy seven professional players, members of three soccer teams, were evaluated for possible participation in the study. The exclusion criteria were as follow: a) any medical disorder that would affect vitamin D levels, b) players that their contract was ending before the conclusion of the study, and c) the consumption of supplements containing vitamin D. Ten (10) players failed to fulfill criteria b (8) and c (2) and were subsequently excluded from the study. Sixty seven Caucasian professional male soccer players, members of two Greek Super league teams (n = 45) and one Football league team (n = 22) were consecutively included in the study. The mean values of age (years) ± SD and height (m) ± SD were: age 25,6±6,2 and height 1,81±0,08 respectively.

### Ethics Statement

Before testing verbal explanation of the aim of the study and the testing procedures were given to all participants, and written informed consent was obtained. The study was performed in strict accordance with the ethical guidelines of the Helsinki Declaration and was approved by the Ethical Scientific Committee of the University Hospital of Heraklion, Crete, Greece.

### Experimental protocol

The duration of the off-season transition period was set to six-weeks, starting at the end of the competition period. During this recuperation period, participants were instructed to avoid any kind of exercise training for the first two weeks. After this two-week period, they were instructed to perform low intensity aerobic running (50%–60% of VO_2_max) of 20 to 30 minute total duration (30, 20, 2×15, 3×10, 2×10) three times per week, divided by one day of rest. This type of activity was selected by the team coaches. All players were tested at two different occasions. Each experimental testing consisted of two days of consecutive measurements. The first experimental testing took place immediately after the end of the competition period in May (pre). The second experimental testing was performed at the beginning of July (post), just prior to the beginning of the preparation period for the forthcoming season. In each experimental periods testing consisted of anthropometric measurements (i.e. height, body weight, body fat percentage), blood testing for the assessment of vitamin D levels, and ergometry tests for the measurement of SJ (cm), CMJ (cm), VO_2_max (ml/kg/min), 10 m, and 20 m sprint performance. The first day of each experimental period anthropometric characteristics were measured at 08∶30 am. From 09∶00 to 10∶30 am, venous blood samples were obtained in order to determine the vitamin D concentration. In the afternoon of the same day (17∶00 pm) the players were tested for SJ, CMJ, 10 m, and 20 m sprint performance. The second day of each experimental period, starting at 09∶30 am, our participants were tested for the determination of VO_2_max. During the study players were instructed to avoid consuming any vitamin D supplements. Before each experimental session, players were informed not to consume any supplement that could promote performance at least 2 days prior to the testing and to avoid any caffeine or alcohol beverages. Moreover, they were instructed to abstain from any exercise training sessions two days prior to each testing in order to avoid any fatigue effects. Detailed nutritional guidelines were given to all players in order to ensure a high (>60%) carbohydrate dietary intake during the study, including a list of a variety of foods, and based on individual resting metabolic rates and the calculated daily energy expenditure as per reported activities (24). Furthermore, players were also asked to maintain their hydration status. All players were familiarized with the testing protocol, as they had been previously tested with the same procedures on several occasions during the last soccer season. The study was performed in Crete, Greece, at latitude of 35,9°N.

### Anthropometric Measurements and Body Composition

Height was measured using a stadiometer (Charder HM210D, Charder Electronics CO, LTD, Taiwan) and weight was obtained using an electronic weight scale (Seca Alpha 770, Seca Vogel, Hamburg, Germany). Body fat percentage was assessed by skinfold thickness measurement (Lange Skinfold Caliper, Cambridge Scientific Instruments, Cambridge, UK) using the 4 site formula by Durnin and Womersley [25].

### Vitamin D measurement

Venus blood samples were obtained following a ten-minute period of rest in a lying position, after an overnight fast. Vitamin D levels were assessed using DiaSorin 25 hydroxy vitamin D (DiaSorin Inc. S.p.A, Italy), repeatability CV = 3–6% and reproducibility CV = 6–11%, according to the laboratory standard operating procedures. Calibrator 1 and 2 (human serum) concentrations are referenced to standard preparations containing highly purified 25(OH) vitamin D. According to the manufacturer the correlation of the immunoenzymatic assay with LC-MS/MS is described by the equation: concentration = 5,6+0,83*LC-MS/MS (R = 0,93). The intra assay coefficient of variation ranges between 3–6%. In our study vitamin D levels below 10 ng/ml were considered as severe deficiency, levels between 10–20 ng/ml as deficiency and levels between 20–30 ng/ml as insufficiency.

### Ergometry tests

The jumping (SJ, CMJ) and sprinting ability (10 m, 20 m) of the soccer players were assessed with a jumping mat (Powertimer, Newtest Ltd., Oulu, Finland) and infrared photoelectric cells (Powertimer, Newtest Ltd., Oulu, Finland) respectively, according to standard procedures [24]. Maximal Oxygen Consumption (VO_2_max) assessment was performed on a motorized treadmill using an automated gas-analysis system (VMAX29, Sensormedics, Yorba Linda, CA), with the use of set procedures of a standard protocol [24].

### Statistical analysis

Statistical analysis was performed using the software program SPSS 17.0. Results are presented as means ± SD. The distribution of variables was tested by the Shapiro-Wilk statistical method. Then, Pearson’s (for normally distributed variables) and Spearman’s (for non-normally distributed variables) correlation coefficients were used to assess the linear relationship between quantative variables. The changes between the experimental periods in the measured parameters within the groups were analyzed by the paired samples t-test for normally-distributed data, and by Mann-Whitney test for non-normally-distributed data. Statistical power analysis was performed (Stata 13 software, StataCorp LP, USA) in order to attain 80% power. Analysis was carried out at a confidence level = 95% and confidence interval = 13,6 [26]. Our calculations showed that a sample size equal to 45, much lower than ours i.e. n = 67, was needed to attain 80% power in order to detect any differences in changes of the measured variables between the two experimental sessions. The level of significance was set at p<0.05.

## Results

### Correlation between vitamin D levels and exercise performance parameters

The correlations between vitamin D levels and exercise performance parameters, during the beginning and the end of the 6-week transition period, are presented in table 1. Analysis of our results revealed a significant positive correlation between vitamin D levels and SJ, CM, and VO_2_max values at the beginning and at the end of the experimental period (table 1). A significant negative correlation was observed between vitamin D levels and 10 m, and 20 m sprint times at the beginning and at the end of the study (table 1).

**Table 1:** Correlations (correlation coefficients and p-values) between Vitamin D levels and exercise performance parameters.

Exercise Performance	Pre Vitamin D (ng/ml)	Post Vitamin D (ng/ml)
SJ (cm)	0.731 (p<0.001)	0.597 (p<0.001)
CMJ (cm)	0.740 (p<0.001)	0.476 (p<0.001)
VO_2_max (ml/kg/min)	0.436 (p<0.001)	0.394 (p = 0.006)
10 m (sec)	−0.649 (p<0.001)	−0.410 (p<0.001)
20 m (sec)	−0.673 (p<0.001)	−0.426 (p<0.001)

Pre: measurement prior to the beginning of the off-season transition period;

Post: measurement at the end of the off-season transition period.

### Changes in vitamin D levels, exercise performance parameters, and body composition

The values of vitamin D and exercise performance parameters at baseline and after the six-week transition period are presented in table 2. Vitamin D levels increased significantly during the off season period compared to baseline (table 2). In contrast, analysis of our data revealed significant reductions in SJ, CMJ, VO_2_max, 10m, and 20m values at the end of the study compared to baseline (table 2). Lastly, there was evident a significant increase in body weight (77,68±7,06 vs 79,08±7,24; p = 0.013) and body fat (8,81±2,96 vs 10,05±3,56; p<0.001) at the end of the study compared to baseline.

**Table 2:** Vitamin D and Performance values in the two experimental periods.

Measurements	Pre	Post
Vitamin D (ng/ml)	34,41±7,08	47,24±13,50**
SJ (cm)	39,50±3,87	37,10±3,59**
CMJ (cm)	40,91±4,57	38,62±4,00**
VO_2_max (ml/kg/min)	59,44±3,07	58,89±3,45**
10 m (sec)	1,74±0,07	1,79±0,08**
20 m (sec)	3,02±0,06	3,07±0,07**

Pre: measurement prior to the beginning of the off-season transition period;

Post: measurement at the end of the off-season transition period.*significant difference at the level of significance p<0.05**, significant difference at the level of significance p<0.01.

## Discussion

Our findings support our hypothesis. Analysis of our results showed that vitamin D levels are associated with neuromuscular performance and aerobic capacity in professional soccer players. Notably, to the best of our knowledge, for the first time our study provides evidence of a linear relationship between vitamin D serum levels, not only with jumping performance, but also with VO_2_max and speed in non-supplemented soccer players. In addition, we have found that even the short off-season period of reduced training stress had a boosting effect on vitamin D levels. Interestingly, this increase in vitamin D levels was evident in parallel to a reduction in aerobic and neuromuscular performance parameters. The latter finding strengthens the well-documented concept that training plays the principal role for exercise adaptations and improvements in exercise performance, whereas all other parameters including vitamin D play a supportive role.

Vitamin D levels exhibited a positive linear relationship with the ergometric evaluation of muscular strength (SJ and CMJ) and speed at both experimental periods. Our observations are comparable to several studies showing that vitamin D is linearly associated with jumping ability and strength in pre-adolescent girls [2] and elderly individuals [7], [12], [27], and in agreement with the observation that 100 m performance was enhanced after a single biodose of ultraviolet radiation in collegiate women [28]. Moreover, a recent vitamin D supplementation-study on professional soccer players revealed that inadequate vitamin D concentration was detrimental to jumping and sprinting ability, whereas supplementation counteracted this effect [29]. Notably, regarding muscular strength, Hamilton et al. [9] reported that vitamin D levels were not associated with lower limb isokinetic muscle function in soccer players. However, this finding was attributed to the different mode of exercise used since, as the authors suggested, vitamin D could preferentially affect muscle groups that were not evaluated in their study. It should be mentioned that both SJ and CMJ are considered to be the most accurate field tests for the determination of the strength levels of the lower limps [23]. In order to perform SJ and CMJ the proximal muscles required are quadriceps, soleus, and gastrocnemius [7]. Those muscles have been found to be predominantly affected by vitamin D deficiency [7]. Furthermore, it is well established that sprint performance is linearly related with both SJ and CMJ [30], [31], suggesting a direct effect of strength levels on sprinting ability. Therefore, based on the aforementioned evidence, our findings indicate a possible effect of vitamin D on jumping ability and strength, which is in turn translated to an affected sprint performance in a similar manner.

The pathways via which vitamin D affects muscular strength (as measured by SJ and CMJ) and sprint performance are still hypothetical. However, there are several potential mechanisms conveying these effects. The ergogenic effects of vitamin D may be related to the regulation of muscle protein synthesis which could affect muscle mass, thanks to the presence of vitamin D receptors on skeletal myocytes [5], [31], [32]. Furthermore, alterations in vitamin D levels also affect its receptors at the expression and activation levels [5], [20], and thus affecting muscle mass [5], [33], neuromuscular coordination [18], and the relative number and the cross-sectional area of type II muscle fibers [16]. Since it well documented that the major determinants of jumping and sprinting ability are muscle strength [31], [34], type II muscle fibers [35], [36] and neuromuscular coordination, [19], [37], any potential effect of vitamin D on these parameters would in turn affect jumping and sprinting capacity in a similar manner.

Analysis of our data revealed a linear association between vitamin D and VO_2_max in both experimental sessions. This finding is supported by an early study which reported increased aerobic capacity as a result of exposure to ultraviolet radiation in collegiate students [8]. Furthermore, a recent study on adolescents observed a positive relationship between vitamin D and aerobic performance on adolescents [38] . Since VO_2_max is related to soccer performance, as indicated by the well-documented relationship between this parameter and the distance covered during a soccer game [15], our findings suggest that in order to perform efficiently during a soccer game optimal vitamin D levels are needed. The observed association between vitamin D levels and VO_2_max could be related to its protective effect on lung function. According to recently published evidence, low vitamin D levels are associated with lower indices of lung function [22] and increased airway reactivity [39]. Since exercise performance and especially aerobic capacity (VO_2_max) is depended on optimal lung function [40], any protective and/or boosting effect of vitamin D on the function of this organ could beneficially influence aerobic performance during exercise [40]. Vitamin D could also influence VO_2_max by affecting iron metabolism and erythropoietin [21], [41]. According to Li and associates [41] vitamin D deficiency results in dysregulation of innate immunity and inflammation which is affecting iron metabolism and contributes to erythropoietin resistance. It is well documented that erythropoietin is linearly associated with changes in red blood cells levels [42]. Thus, vitamin D could influence VO_2_max via its effects on erythropoesis modifying the capacity of the oxygen supply to the exercising muscles and consequently affecting aerobic exercise performance [42].

Interestingly, analysis of our data showed that although vitamin D increased at the end of the off-season period all measured exercise performance parameters decreased. However, the observed linear correlation at baseline between vitamin D levels and performance was retained at the end of the study, suggesting that vitamin D is related to the ability to perform efficiently during exercise, irrespective the level of performance. These data clearly suggest that although vitamin D does seem to affect neuromuscular and aerobic performance, it does not play the primary role. Indeed, during periods of reduced training stimulus or training cessation (i.e. soccer transition period) there is deterioration in exercise performance [24]. Since this decline is widely accepted to be a result of the insufficient training stimulus [24], this finding demonstrates that the major determinant of exercise performance is the amount and the quality of training [15], [24]. The latter evidence documents that vitamin D plays a supportive role in exercise performance. However, this does not lessen the importance of vitamin D serum concentration since in elite soccer level even subtle changes in performance may determine the outcome of a competition.

In our study the six-week transition period had a boosting effect on vitamin D levels. Indeed, at the first experimental period, although none of our participants was vitamin D deficient (<20 ng/ml) or severe deficient (<10 ng/ml), 55,22% of our players had insufficient vitamin D levels (<30 ng/ml), whereas at the second one only 4,47% were found to be below 30 ng/ml. In regard to the first experimental session our findings are in agreement with studies on soccer players (∼50% <30 ng/ml) [43], and members of the American national football league [44] (∼51% <30 ng/ml). Furthermore, our values were at the lower level of the range observed (30–84% vitamin D insufficiency) in several athletic and non-athletic population [9], [43], [45]. The most plausible explanation for the elevation of vitamin D levels at the second experimental session could be the consequence of an increased exposure to UVB during the off-season period [9]. Indeed, this transition period in Greek Superleague takes place during June and at the beginning of July at a favorable latitude (35,9°N). During this period UVB reaches its peak [46], resulting in increased vitamin D production. It is well demonstrated the extremely importance that UVB plays on vitamin D synthesis [1], [3] and the observation that its effectiveness is among other parameters, season dependent [32]. Furthermore, this off-season phase is actually a holiday period for professional soccer players. This could indicate increased time spend under sunlight, and also an increased exposure of a larger proportion of the players’ body to UVB. Since all these parameters are related with vitamin D production, the observed increased in its levels at the end of our study could be partly accounted to these factors [3], [47]. The above mentioned suggestions are further supported by the observation that 84% of 342 Qatar soccer players were vitamin D insufficient (<30 ng/ml) [9], despite of the favorable latitude (25.4°N) and the period that the study was performed (i.e. July). This finding was attributed to inadequate exposure to the sun, since all outdoors training were performed after sunset, demonstrating the importance of UVB on vitamin D production.

Although our data reaffirm the importance of adequate exposure to UVB, recent evidence indicate that the exercise training -induced stress may play a regulatory role on vitamin D levels [14]. Andersen et al. [14] reported that intense military training resulted to reduced vitamin D levels in female soldiers, although these activities were performed outdoors in the summer and early autumn. This finding was unanticipated by the authors since they expected vitamin D levels to increase or at least to remain constant due to the adequate daily exposure to UVB during the whole study. Further support is coming from two recent studies on soccer players [43], [48]. The authors observed much lower vitamin D levels (30,82±9,04 [42], 32,83±6,64 [48]) and insufficiency values (83% <30 ng/ml [42], 65% <30 ng/ml [48]) during periods of training, compared to the ones we obtained after the six-week detraining period (45,67±13,70; 4,47<30 ng/ml). These low values were evident despite the fact that outdoor training sessions were performed at August [48] and during summer [43] under a sufficient daily exposure of a large proportion of their body to UVB. According to Holick [49], the participants of these two studies should have had sufficient vitamin D levels since the amount of sun exposure needed to maintain adequate vitamin D levels is approximately 5–30 minutes at least twice a week to the face, arms, and legs [49]. Based on the aforementioned evidence we could suggest that the exercise training-induced stress could play a regulatory role on vitamin D levels. Supporting to our hypothesis is the fact that our second testing was performed following a massive reduction of training stress, while the participants in these soccer studies were tested in pre-season and early in-season which are periods of high training stress (i.e. training sessions and games) [15]. This suggested impact of exercise-induced stress on vitamin D could be associated to the immune effects of the intense stress [50], since both vitamin D and exercise training are related with the immune system [50], [51]. Indeed, prolonged intense training sessions or intensified training periods, similar to the ones used repeatedly during a soccer season, suppresses athletes’ immune system [50]. In our study, the first experimental session was performed at the end of competition season which according to the literature suppresses innate immunity [50]. On the contrary, the six-week period prior to the second testing our players were under minimal training stress, which could hypothetically have resulted in an enhanced innate immunity. Recently published data suggest a strong inverse correlation between inflammation and vitamin D concentration [8], [51], while elevated vitamin D levels boost immunity [52]. Therefore, we could hypothesize that any positive alteration in soccer players’ immunity due to reduced training stress could be evident in conjunction with increased vitamin D status.

Our study provides evidence of an association between not supplemented vitamin D levels and parameters of aerobic and neuromuscular exercise performance in soccer. In particular, our findings indicated a linear relationship between vitamin D levels and muscle strength as evaluated by SJ and CMJ, sprinting ability (10 m, and 20 m), and VO_2_max in professional soccer players. Moreover, our results indicate that, apart from increased exposure to UVB, reductions in exercise training stress may also have beneficial effects on vitamin D levels in elite soccer players. However in order to confirm this hypothesis additional research is needed, also examining indices of inflammation. Furthermore, our findings indicate that vitamin D plays, among other parameters, a secondary supportive role in athletic performance. However, this does not lessen its importance, especially in highly competitive athletes, since in elite athletic level slight changes in performance may determine the outcome of a competition. Our current data may provide an additional tool to sport scientists, coaches, and players to enhance soccer performance.

## Supporting Information

File S1 **Supplementary File Data.xlsx which include all performed vitamin D and performance parameters measurements in this study.**
(XLSX)
